# Evaluating Radiotheranostic Targets for Endometrial Cancer

**DOI:** 10.2967/jnumed.125.270318

**Published:** 2025-10

**Authors:** Joni Sebastiano, Shane A. McGlone, Zachary V. Samuels, Camilla Grimaldi, Ava Stoddard, Sugar Galka, Emma Colaco, Brian M. Zeglis

**Affiliations:** 1 Department of Chemistry, Hunter College, City University of New York, New York, New York;; 2 Department of Radiology, Memorial Sloan Kettering Cancer Center, New York, New York;; 3 PhD Program in Biochemistry, Graduate Center of City University of New York, New York, New York;; 4 PhD Program in Chemistry, Graduate Center of City University of New York, New York, New York;; 5 PhD Program in Biology, Graduate Center of City University of New York, New York, New York; and; 6 Department of Radiology, Weill Cornell Medical College, New York, New York

**Keywords:** radiopharmaceuticals, CD24, HER2, MUC16, endometrial cancer, PET

## Abstract

Endometrial cancer is the most common gynecologic malignancy worldwide, and its incidence and mortality rates have increased over the past decade. Although early-stage disease is effectively treated via hysterectomy, a dearth of molecularly targeted therapies means that prognoses are far poorer for those with disseminated or recurrent disease. Herein, we describe the exploration of 3 biomarkers—human epidermal growth factor receptor 2 (HER2), mucin-16 (MUC16), and CD24—as potential radiotheranostic targets for endometrial cancer. **Methods:** Immunocytochemical staining was used to evaluate the expression of HER2, MUC16, and CD24 in endometrial cancer and healthy uterine and healthy endometrial cell lines. Subsequently, similar immunofluorescence staining analyses were performed with patient-derived endometrial cancer, healthy cervix, and healthy endometrium tissue samples. Stochastic bioconjugation methods were used to append deferoxamine (DFO) to a trio of monoclonal antibodies: trastuzumab (αHER2), AR9.6 (αMUC16), and ATG-031 (αCD24). These immunoconjugates were labeled with [^89^Zr]Zr^4+^, and the in vivo performance of the resulting immuno-PET probes—[^89^Zr]Zr-DFO-trastuzumab, [^89^Zr]Zr-DFO-AR9.6, and [^89^Zr]Zr-DFO-ATG-031—was interrogated via PET imaging and biodistribution experiments in cell line and patient-derived murine models of endometrial cancer. **Results:** Immunofluorescent staining revealed that endometrial cancer cells and tissue samples expressed elevated levels of HER2, MUC16, and CD24 compared with healthy control cells and tissue samples. [^89^Zr]Zr-DFO-trastuzumab, [^89^Zr]Zr-DFO-AR9.6, and [^89^Zr]Zr-DFO-ATG-031 were synthesized with high radiochemical conversion (>95%), radiochemical purity (>95%), specific activity (4–5 mCi/mg), and immunoreactivity (>80%). The 3 immuno-PET probes exhibited significantly different behavior in mice bearing subcutaneous endometrial cancer xenografts: [^89^Zr]Zr-DFO-ATG-031 provided the highest tumor uptake (>30 %ID/g) and tumor-to-background contrast; [^89^Zr]Zr-DFO-trastuzumab produced moderate yet promising results; and [^89^Zr]Zr-DFO-AR9.6 yielded substandard images. Subsequent imaging experiments in mice bearing patient-derived xenografts reinforced the potential of the CD24- and HER2-targeted immuno-PET probes. **Conclusion:** [^89^Zr]Zr-DFO-ATG-031 and [^89^Zr]Zr-DFO-trastuzumab exhibit significant promise for immuno-PET imaging of endometrial cancer. These probes may prove beneficial in the clinic, not only for the noninvasive staging and monitoring of the disease but also as companion imaging agents for HER2- and CD24-targeted therapeutics.

## Visual Abstract

**Figure:**
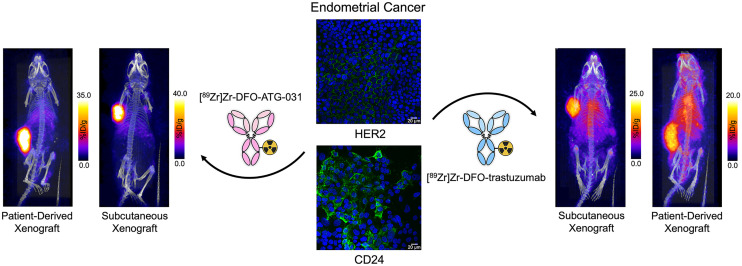

Endometrial cancer, a malignancy of the inner epithelial lining of the uterus, is the most common gynecologic cancer worldwide. The incidence of endometrial cancer is rising 1%–2% every year, and it is one of the few cancers whose mortality rate is increasing (1). Although the median patient age at diagnosis is 61 y, endometrial cancer is increasingly found in younger women: cases in women younger than 40 y have doubled in recent years (2). Rising global rates of obesity may be partially responsible for this phenomenon, as obesity has been linked to both higher rates of endometrial cancer as well as poor prognoses (1). Despite the ubiquity of the disease, there are also alarming racial disparities in both the diagnosis and mortality of endometrial cancer. In the United States, Black women are twice as likely to be diagnosed with and die of endometrial cancer compared with women of other races (3).

The diagnosis of endometrial cancer typically relies on pelvic ultrasound and dilation and curettage. As the former has a relatively low specificity for the disease, a biopsy via dilation and curettage is commonly required for patients showing symptoms, such as postmenopausal bleeding or pelvic pain (4). However, only about 10% of patients with these symptoms are diagnosed with the malignancy, so less invasive detection methods are frequently cited as a clinical need (1,5). Early-stage endometrial cancer is typically treated via hysterectomy and bilateral salpingo-oophorectomy followed by adjuvant brachytherapy, external beam radiotherapy, or systemic chemotherapy (1). Although this paradigm is largely effective (survival rates exceed 80% for stage 1 and 2 disease), complete hysterectomies increase patients’ risk for a variety of morbidities, including heart disease and stroke, and this treatment strategy is obviously unsuitable for patients seeking to preserve their fertility (6). The situation is much more dire for patients diagnosed with regional or distant disseminated disease: the 5-y overall survival rates for patients with stage 3 and stage 4 disease are 52% and 15%, respectively. Furthermore, recurrent disease occurs in 10%–20% of patients with early-stage disease who have been treated surgically; in these unfortunate patients, the 5-y overall survival rates for local and metastatic recurrences are 55% and 17%, respectively (7,8). In recent years, a variety of molecularly targeted therapies—including hormone-targeted and vascular endothelial growth factor–targeted treatments—have been explored in the context of endometrial cancer but have produced only middling results (8,9). Most optimistically, both pembrolizumab and dostarlimab have been approved for the checkpoint inhibitor therapy of patients with advanced or recurrent endometrial cancer, but response rates are variable and highly sensitive to the molecular subtype of the disease (10,11).

The past 2 decades have been witness to a remarkable surge in the use of radiopharmaceuticals for the imaging and therapy of cancer. The 2 standard bearers of this movement, [^177^Lu]Lu-PSMA-617 and [^177^Lu]Lu-DOTATATE, have proven highly effective for the treatment of prostate cancer and neuroendocrine tumors, respectively, and have inspired a renaissance in radiopharmaceutical therapy that has embraced an ever-expanding array of targets, platforms, and radionuclides (12). In parallel, the value of nuclear imaging—PET in particular—as a theranostic tool has come into focus. PET is increasingly deployed not only for diagnosis and staging but also to identify patients likely to respond to molecularly targeted therapies, including antibody–drug conjugates, checkpoint inhibitors, and radiotherapeutics (13,14). [^18^F]FDG PET has been used in the context of endometrial cancer, but very few molecularly targeted PET probes and radiotherapeutics have been explored for the disease (8). In 2013, Fu et al. reported on the use of a ^64^Cu-labeled epithelial membrane protein-2 minibody as an imaging agent for endometrial cancer, although the tracer exhibited significant uptake in healthy tissues, and the xenograft model used cells transfected to overexpress the target (15). The overall lack of work in this area is surprising, as radiopharmaceuticals could meet several urgent needs: nuclear imaging agents could aid in initial staging, postsurgical monitoring, and the selection of patients for targeted treatments, while radiotherapeutics could improve outcomes for patients with disseminated, metastatic, and recurrent disease.

We report on the evaluation of 3 antigens—human epidermal growth factor receptor 2 (HER2), mucin-16 (MUC16), and CD24—as potential radiotheranostic targets in endometrial cancer. HER2 is a transmembrane receptor most often associated with breast cancer but is overexpressed in a host of other malignancies; MUC16 is a type I transmembrane mucin most often associated with ovarian and pancreatic cancer; and CD24 is a small, membrane-bound glycoprotein that is overexpressed in several solid carcinomas, such as esophageal squamous cell carcinoma and ovarian cancer (16). Each has emerged as a therapeutic target for antibody–drug conjugates and immunotherapeutics (17,18). Furthermore, all 3 have been identified as potential markers of endometrial cancer, although—critically—none have been exploited for the nuclear imaging or radiopharmaceutical therapy of the disease (19). Herein, we describe our work to assess the radiotheranostic potential of this trio of targets. Specifically, we used cell lines and patient-derived tissue samples to interrogate the expression of each target in endometrial cancer, the healthy endometrium, and the healthy uterus; synthesized and characterized ^89^Zr-labeled radioimmunoconjugates based on the monoclonal antibodies (mAb) trastuzumab (αHER2), AR9.6 (αMUC16), and ATG-031 (αCD24); and evaluated the in vivo performance of these immuno-PET probes in cell line–derived and patient-derived murine models of endometrial cancer.

## MATERIALS AND METHODS

All reagents were purchased from Fisher Scientific unless otherwise noted. Trastuzumab and AR9.6 were provided by the Radiochemistry and Molecular Imaging Probes Core of Memorial Sloan Kettering Cancer Center, and ATG-031 was purchased from MedChemExpress. Protein concentrations were determined via ultraviolet–visible spectroscopy using a molar absorptivity of 2.1 × 10^5^ M^−1^ cm^−1^ at 280 nm and a molecular weight of 1.5 × 10^5^ Da. All water used was ultrapure (>18.2 MΩ·cm at 25 °C). *p*-SCN-Bn-desferrioxamine (DFO) was purchased from Macrocyclics. Matrix-assisted laser desorption ionization mass spectrometry was performed by the Alberta Proteomics and Mass Spectrometry Facility (University of Alberta). ^89^Zr was provided by 3D Imaging. Additional methods and materials are detailed in the supplemental materials, available at http://jnm.snmjournals.org (20,21).

### Immunocytochemistry and Confocal Imaging

Human endometrial cancer (HEC-1-A) cells were seeded (5 × 10^4^) in triplicate into chamber slides (ThermoFisher) and left to adhere overnight. Cells were fixed using neutral buffered 10% formalin (20 min at room temperature [RT]), blocked with phosphate-buffered saline plus 5% animal serum (1 h at RT), and washed 3 times with phosphate-buffered saline between each step. Immunofluorescence staining was performed via incubation with a 5 µg/mL solution of trastuzumab, AR9.6, or ATG-031 (1 h at RT). After 3 washes with phosphate-buffered saline, a secondary antibody solution (Alexa Fluor 488–conjugated goat antihuman IgG plus 4′,6-diamidino-2-phenylindole) was added to the samples (1 h at RT). A set of slides for each cohort did not receive the primary antibody but did receive the secondary antibody to serve as a negative control. Cover slips were then added to the chamber slides using Fluoroshield histology mounting medium (Sigma-Aldrich), and the slides were imaged via confocal microscopy. Confocal images were analyzed using ImageJ, and histograms were kept consistent between each sample.

### Staining of Patient-Derived Tissue Samples

Patient-derived endometrial adenocarcinoma tissue was obtained from the Anti-Tumor Assessment Core at Memorial Sloan Kettering Cancer Center. Healthy human endometrial and cervical tissue slides were obtained from Tissue Array. All tissue specimens received were formalin-fixed paraffin-embedded before arrival. Paraffin was removed from the tissue samples using xylenes followed by serial dilutions with ethanol and water. Antigen retrieval was performed using a trypsin antigen retrieval kit (AbCam) in accordance with the manufacturer-provided protocol. Immunofluorescent staining of the tissue samples was performed using the same procedure as described above. Confocal images were analyzed using ImageJ, and histograms were kept consistent between each sample, including a negative control that did not receive the primary antibody.

### Subcutaneous Xenografts

All animal care was approved by the institutional animal care and use committees of Hunter College and Weill Cornell Medical College. Female athymic nude mice (5- to 8-wk old; The Jackson Laboratory) were allowed to acclimatize for 1 wk before inoculation. The animals were housed in ventilated cages and given food and water ad libitum. The mice were anesthetized by inhalation of 2% isoflurane–oxygen gas mixture (Baxter Healthcare), and the injection site was sanitized with an ethanol wipe. Tumors were induced in the right shoulder via the subcutaneous injection of 2 × 10^6^ HEC-1-A cells in a 1:1 mixture of medium:MatriGel (Corning Life Sciences). Tumors reached an acceptable size for PET imaging and biodistribution studies (100–200 mm^3^) after approximately 4 wk.

### PET Imaging

PET images were acquired using an Inveon PET/CT small-animal scanner (Siemens Medical Solutions). After the subcutaneous tumors reached an appropriate size for experimentation, the mice were placed under a heat lamp to facilitate vein dilation, and their skin was sterilized using an ethanol wipe. [^89^Zr]Zr-DFO-trastuzumab, [^89^Zr]Zr-DFO-AR9.6, or [^89^Zr]Zr-DFO-ATG-031 [3.7 MBq (100 μCi)] (*n* = 4 per cohort) was administered via tail-vein injection, and static PET/CT scans were acquired at 24, 72, and 120 h after injection. The counting rates in the reconstructed images were converted to activity concentrations (percentage injected dose per gram of tissue [%ID/g]) using a system calibration factor derived from the imaging of a mouse-sized water-equivalent phantom containing ^89^Zr. Maximum-intensity-projection (MIP) images were generated from 3-dimensional ordered-subset expectation maximum reconstruction. The images were analyzed with VivoQuant (Invicro).

### Biodistribution Studies

After the final imaging time point (120 h after injection), the animals were euthanized via asphyxiation with carbon dioxide gas followed by cervical dislocation. Blood, tumor tissue, and selected organs were harvested, rinsed in water, dried, and weighed, and the amount of activity in each was quantified on a ^89^Zr-calibrated automatic Wizard2 γ-counter (PerkinElmer). The counts per min for each tissue was background- and decay-corrected to the start of the activity measurement. The %ID/g value for each sample was then calculated by normalization to the mass of the tissue and the total injected activity.

### Patient-Derived Xenografts (PDXs)

PDX samples were provided by the laboratory of Britta Weigelt at the Memorial Sloan Kettering Cancer Center. The tumors were implanted into the right flank of NSG (NOD.Cg-Prkdcscid) mice by the Memorial Sloan Kettering Antitumor Assessment Core in accordance with protocols approved by the institutional animal care and use committee.

## RESULTS

### Evaluating the Expression of HER2, MUC16, and CD24 in Endometrial Adenocarcinoma

**FIGURE 1.**
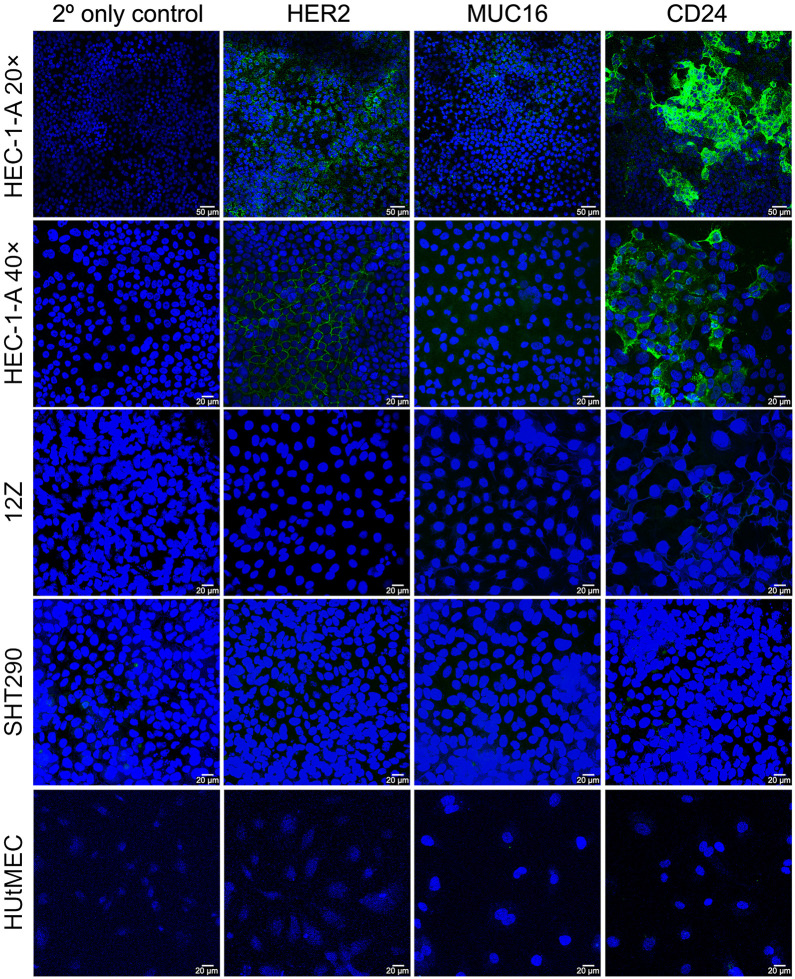
Immunocytochemical staining of HEC-1-A (×20 and ×40 magnifications), 12Z, SHT290, and human uterine microvascular endothelial cells (HUtMEC) for HER2, MUC16, and CD24. Alexa Fluor 488–bearing secondary antibodies were used for antigen staining (green), and 4′,6-diamidino-2-phenylindole was used to stain DNA (blue).

Immunocytochemical staining was used to evaluate the expression levels of HER2, MUC16, and CD24 in HEC-1-A cells. To this end, immobilized HEC-1-A cells were incubated with trastuzumab (αHER2), AR9.6 (αMUC16), or ATG-031 (αCD24) and subsequently stained with a fluorophore-modified antihuman secondary antibody. Immunofluorescence imaging clearly revealed that the cells expressed the 3 antigens to varying degrees, with the MUC16 cells exhibiting the least pronounced and the CD24 cells showing the most intense expression (Fig. 1). To interrogate the expression of these 3 antigens in the endometrium and uterus, we also performed immunocytochemical staining with 3 healthy cell lines: endometrial epithelial cells (12Z), endometrial stromal cells (SHT290), and human uterine microvascular endothelial cells. Critically, HER2, MUC16, and CD24 were either absent or expressed at very low levels in each of these healthy cell lines.

**FIGURE 2.**
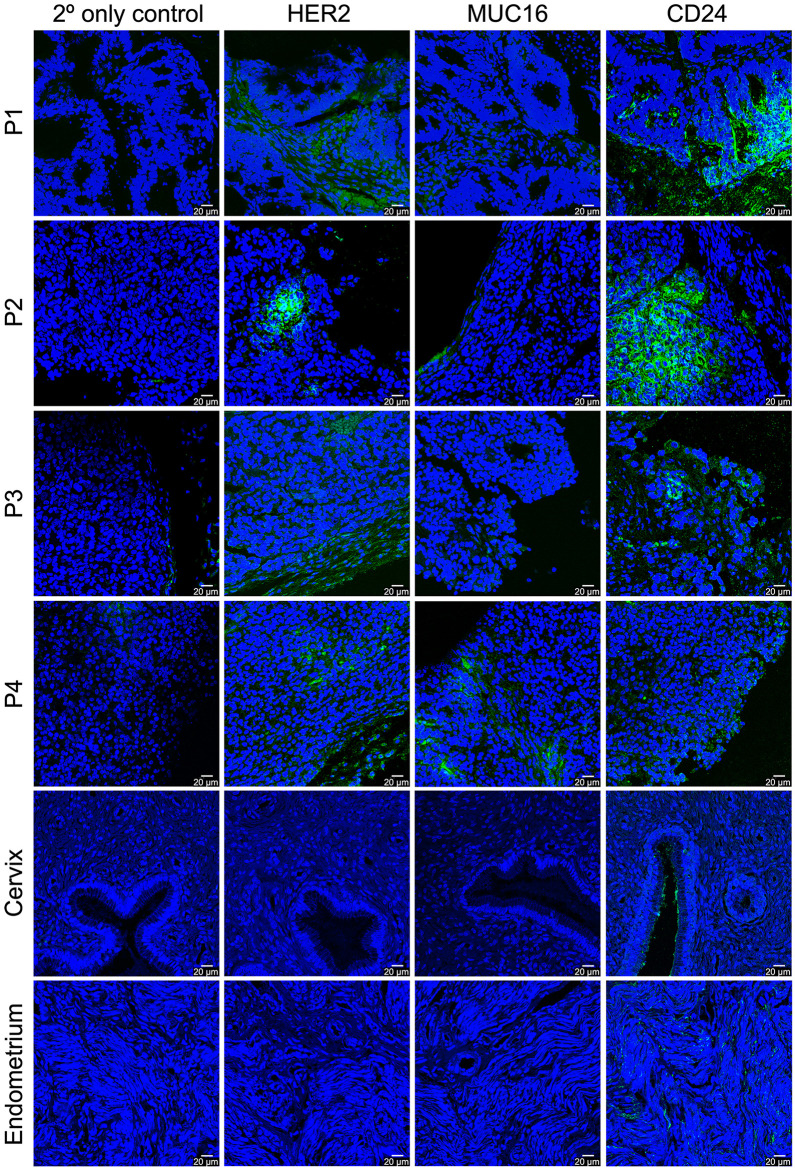
Histologic immunofluorescence staining of patient-derived endometrial adenocarcinoma, healthy cervix, and healthy endometrium tissue samples for HER2, MUC16, and CD24. Alexa Fluor 488–bearing secondary antibodies were used for antigen staining (green), and 4′,6-diamidino-2-phenylindole was used to stain DNA (blue).

We next characterized the expression of these antigens in patient-derived endometrial cancer tissue samples obtained from the Memorial Sloan Kettering Antitumor Assessment Core. To this end, 4 different tumor samples of various molecular subtypes of the disease—microsatellite instability (P1), copy-number low (P2), and polymerase epsilon (POLE) ultramutated (P3), and P4 (subtype unknown)—were incubated with trastuzumab, AR9.6, or ATG-031; stained with a fluorophore-labeled secondary mAb, and evaluated via confocal microscopy (Fig. 2) (22,23). All 4 samples displayed consistent and abundant expression of HER2 and CD24, with samples of P1 and P2 displaying elevated levels compared with P3 and P4. The expression of MUC16 was more variable, with P1, P2, and P3 samples exhibiting low levels of the mucin, but the P4 sample containing higher levels of the protein. Importantly, parallel staining was performed using tissue samples of healthy human endometrium and healthy human cervix. Neither tissue showed appreciable expression of HER2 or MUC16; however, both displayed levels of CD24 that were detectable yet significantly below those evident in the tumor samples.

### Synthesizing HER2-, MUC16-, and CD24-Targeted ^89^Zr-Immuno-PET Probes

**FIGURE 3.**
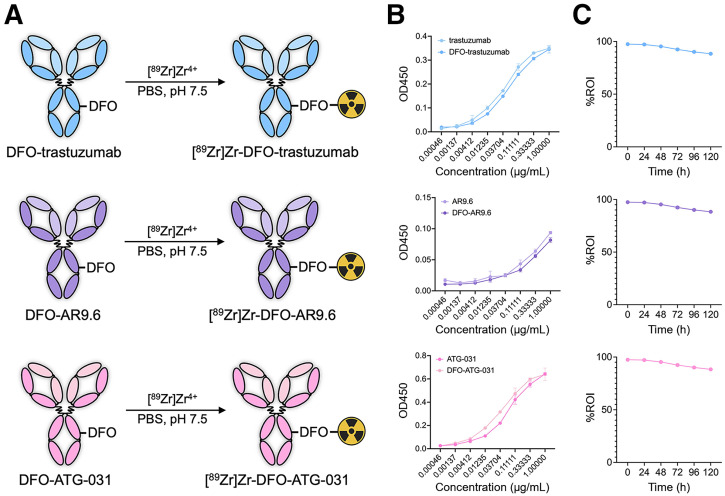
(A) ^89^Zr-labeling of DFO-trastuzumab, DFO-AR9.6, and DFO-ATG-031. (B) Enzyme-linked immunosorbent assay binding curves for DFO-trastuzumab, DFO-AR9.6, and DFO-ATG-031 compared with their native counterparts. (C) Results of human serum stability over 5 d for [^89^Zr]Zr-DFO-trastuzumab, [^89^Zr]Zr-DFO-AR9.6, and [^89^Zr]Zr-DFO-ATG-031 obtained via radio-instant thin-layer chromatography. PBS = phosphate-buffered saline; OD450 = optical density at 450 nm; ROI = relative optical intensity.

Trastuzumab, AR9.6, and ATG-031 were stochastically modified with the chelator DFO via the modification of lysine residues using *p*-SCN-Bn-DFO. The resulting immunoconjugates—DFO-trastuzumab, DFO-AR9.6, and DFO-ATG-031—were obtained in a yield greater than 90% after purification via gel filtration chromatography, and size-exclusion high-performance liquid chromatography revealed that bioconjugation did not result in the aggregation or fragmentation of the mAb (Supplemental Fig. 1). In addition, the degrees of labeling of DFO-trastuzumab, DFO-AR9.6, and DFO-ATG-031 were determined to be 2.4 ± 0.2, 2.6 ± 0.1, and 2.7 ± 0.1 DFO/mAb, respectively, using matrix-assisted laser desorption ionization–time-of-flight mass spectrometry (Supplemental Fig. 2). Finally, enzyme-linked immunosorbent assays using recombinant antigens confirmed that the modification of each mAb did not affect its ability to bind its biomolecular target (Fig. 3B).

The trio of immunoconjugates was subsequently radiolabeled with [^89^Zr]Zr^4+^ using standard published procedures, yielding [^89^Zr]Zr-DFO-trastuzumab, [^89^Zr]Zr-DFO-AR9.6, and [^89^Zr]Zr-DFO-ATG-031 (Fig. 3A). All 3 radioimmunoconjugates were synthesized with high radiochemical conversion (>95%), purity (>99%) after gel filtration chromatography, and specific activities (185–370 MBq/mg [5–10 mCi/mg]) (Supplemental Fig. 3). Size-exclusion high-performance liquid chromatography confirmed that the radiolabeling process did not result in any aggregation or fragmentation of the immunoglobulins, and stability assays showed that each ^89^Zr-labeled mAb remained over 85% intact to demetallation after 5 d in human serum at 37 °C (Fig. 3C). Bead-based binding assays revealed that [^89^Zr]Zr-DFO-trastuzumab and [^89^Zr]Zr-DFO-ATG-031 boasted immunoreactive fractions of 0.88 ± 0.01 and 0.90 ± 0.01, respectively (Supplemental Fig. 4). In the case of [^89^Zr]Zr-DFO-AR9.6, however, a bead-based assay was impossible because of the nature of MUC16. Instead, a cell-based binding assay with HEC-1-A cells provided an immunoreactive fraction of 0.15 ± 0.02, a result that likely stems from the meager expression of the antigen in this cell line rather than any problem with the radioimmunoconjugate itself.

### Exploring the Performance of the ^89^Zr-mAb in Murine Models of Endometrial Cancer

**FIGURE 4.**
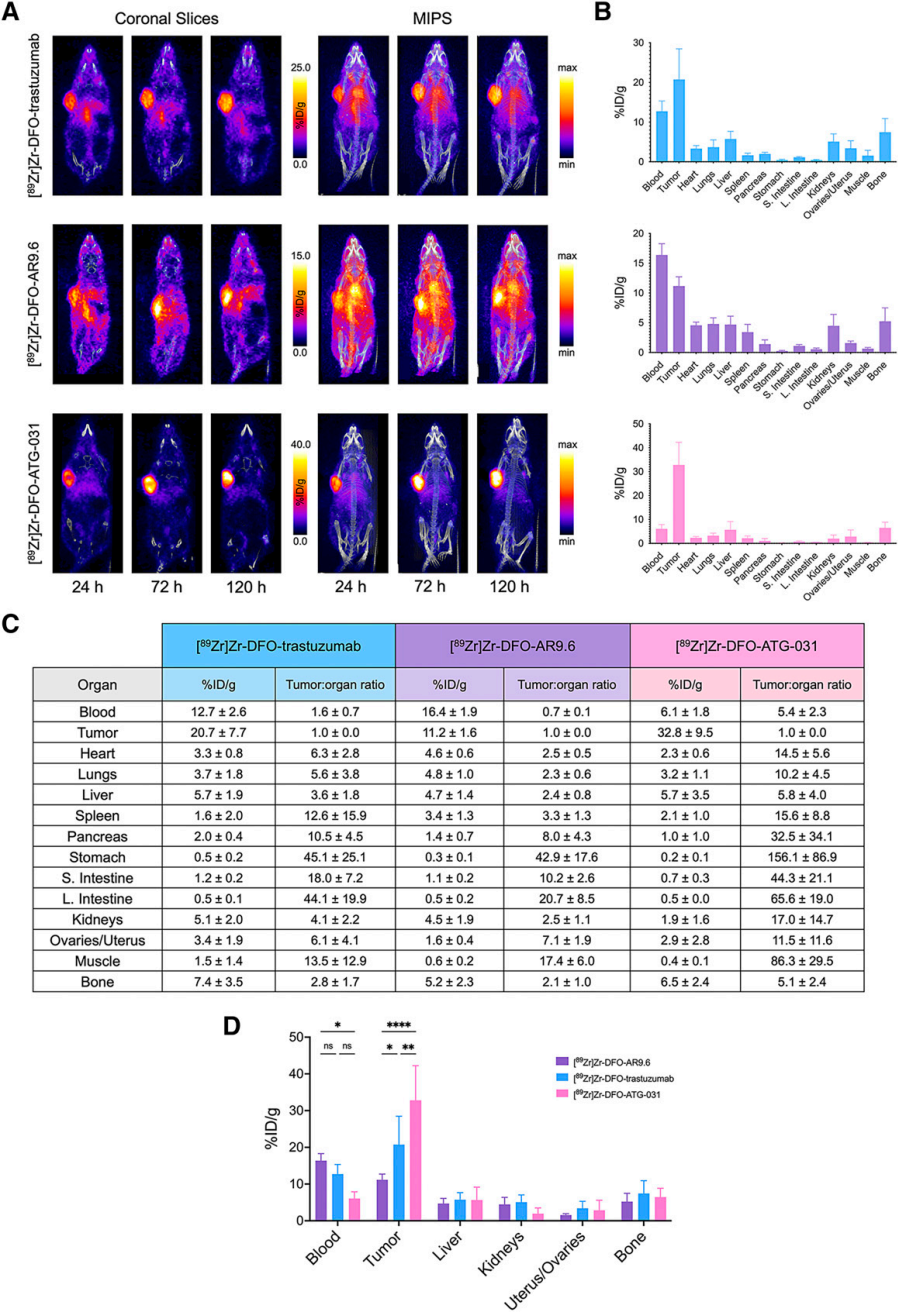
(A) Coronal slices and maximum-intensity projections (MIPS) of athymic nude mice bearing subcutaneous HEC-1-A xenografts acquired 24, 72, and 120 h after intravenous administration of [^89^Zr]Zr-DFO-trastuzumab, [^89^Zr]Zr-DFO-AR9.6, or [^89^Zr]Zr-DFO-ATG-031. (B) Biodistribution data collected after final imaging time point. (C) Table of quantitative biodistribution data (%ID/g) and tumor-to-background organ activity concentration ratios. (D) Comparison of biodistribution data from selected organs. *P < 0.05; **P < 0.01; ****P < 0.0001; ns = not significant.

The in vivo evaluation of [^89^Zr]Zr-DFO-trastuzumab, [^89^Zr]Zr-DFO-AR9.6, and [^89^Zr]Zr-DFO-ATG-031 was first performed in mice bearing subcutaneous HEC-1-A endometrial cancer xenografts. To this end, tumor-bearing mice were administered [^89^Zr]Zr-DFO-trastuzumab, [^89^Zr]Zr-DFO-AR9.6, or [^89^Zr]Zr-DFO-ATG-031 (3.7–3.9 MBq; 20–21 µg; in 100 µL of 0.9% sodium chloride solution) via the tail vein. PET images were subsequently acquired at 24, 72, and 120 h after injection, and biodistribution data were collected after the final imaging time point. Although all of the radioimmunoconjugates delineated the tumor tissue, they did so with varying tumor-to-background contrast (Fig. 4). [^89^Zr]Zr-DFO-AR9.6 exhibited accretion in the tumor, but it was relatively meager (11.2 ± 1.6 %ID/g at 120 h after injection) and accompanied by high background signal (16.4 ± 1.9 %ID/g in the blood at the same time point). [^89^Zr]Zr-DFO-trastuzumab, in contrast, displayed significantly higher uptake in tumor tissue (20.7 ± 7.7 %ID/g at 120 h after injection) and substantially better tumor-to-background contrast (i.e., tumor-to-reproductive organs activity concentration ratio of 6.1 ± 1.9 at 120 h after injection). Finally, [^89^Zr]Zr-DFO-ATG-031 exhibited the best performance by far, producing high tumoral activity concentrations (32.8 ± 9.5 %ID/g) and excellent tumor-to-background activity concentration ratios—approximately 5, 12, and 66 for the blood, uterus and ovaries, and large intestine, respectively—at 120 h after injection.

We next turned to a PDX model to interrogate the performance of these radioimmunoconjugates in a more realistic recapitulation of human disease. To this end, NSG mice were inoculated with PDX samples from a patient with copy-number low endometrial adenocarcinoma (P2). Considering the suboptimal performance of [^89^Zr]Zr-DFO-AR9.6 in mice bearing subcutaneous xenografts and the lack of MUC16 expression in the P2 sample, only [^89^Zr]Zr-DFO-trastuzumab and [^89^Zr]Zr-DFO-ATG-031 were evaluated in the PDX model. Critically, we previously demonstrated that the interaction between radioimmunoconjugates and the unoccupied Fc-γ-receptor 1 of highly immunocompromised mice results in anomalous biodistribution profiles characterized by high uptake in the liver and spleen (24). To circumvent this issue, we deglycosylated DFO-trastuzumab and DFO-ATG-031 with peptide *N*-glycosidase F, as the removal of the heavy-chain glycans prompts a conformational shift in the immunoglobulin that blocks interactions with Fc-γ-receptor 1. The success of the deglycosylation was confirmed via sodium dodecyl sulfate–polyacrylamide gel electrophoresis (Supplemental Fig. 5).

**FIGURE 5.**
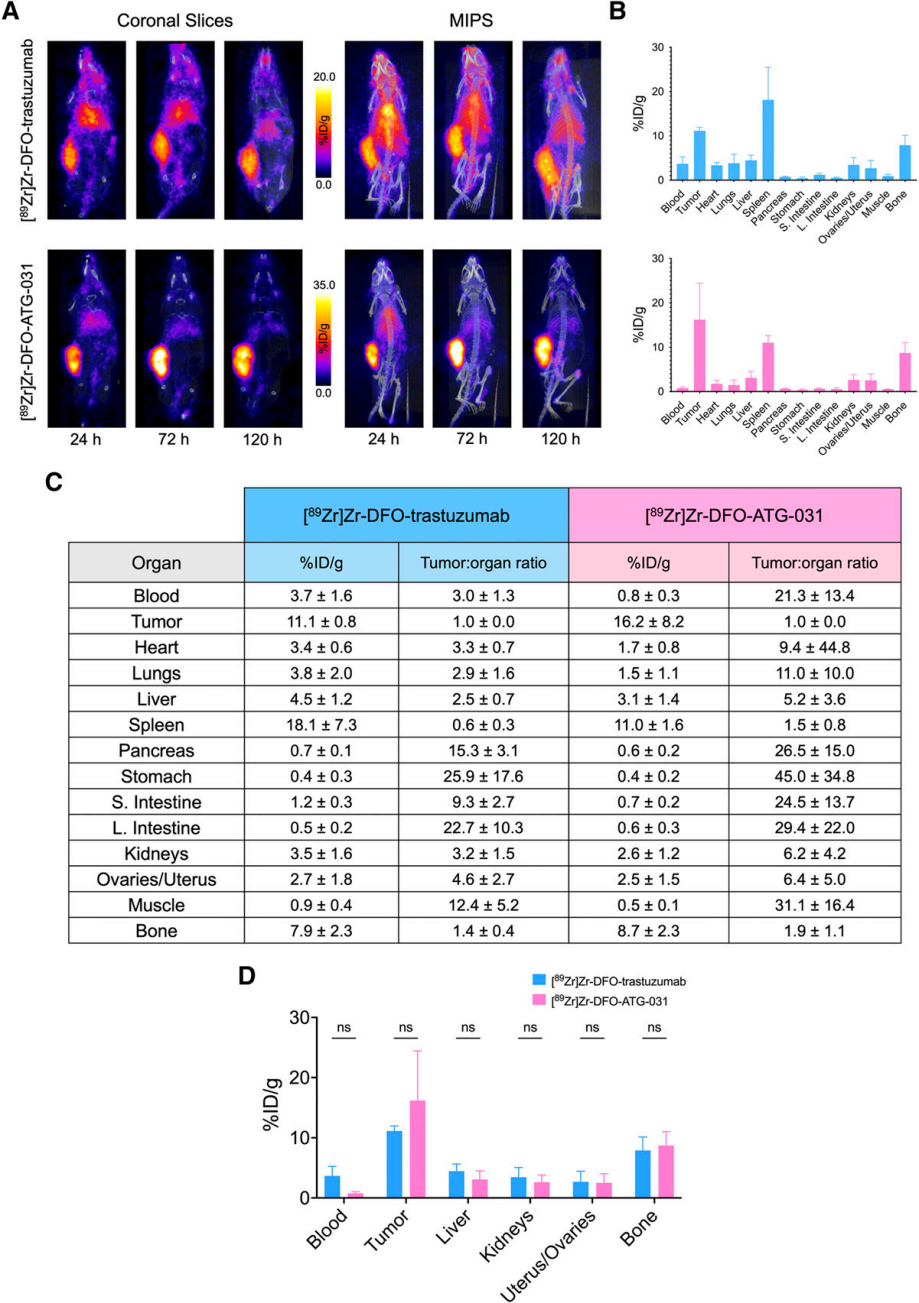
(A) Coronal slices and maximum-intensity projections (MIPS) of NSG mice bearing subcutaneous PDXs collected 24, 72, and 120 h after intravenous administration of [^89^Zr]Zr-DFO-trastuzumab or [^89^Zr]Zr-DFO-ATG-031. (B) Biodistribution data collected after final imaging time point. (C) Table of quantitative biodistribution data (%ID/g) and tumor-to-background organ activity concentration ratios. (D) Comparison of biodistribution data from selected organs. ns = not significant.

The 2 deglycosylated immunoconjugates were radiolabeled with [^89^Zr]Zr^4+^ to yield [^89^Zr]Zr-DFO-^degly^trastuzumab and [^89^Zr]Zr-DFO-^degly^ATG-031 in high yield (>95%), purity (>98%), and specific activity (185–370 MBq/mg [5–10 mCi/mg]). Subsequently, each was injected into NSG mice bearing subcutaneous PDXs via the lateral tail vein (3.7–3.9 MBq; 20–21 µg; in 100 µL of 0.9% sodium chloride solution). PET images were collected 24, 72, and 120 h thereafter, and biodistribution data were acquired after 120 h. The behavior of the ^89^Zr-labeled mAb in this model largely mirrored its performance in the mice bearing HEC-1-A tumors. [^89^Zr]Zr-DFO-trastuzumab produced moderate uptake in tumor tissue (11.1 ± 0.8 %ID/g at 120 h after injection) and moderate yet promising tumor-to-background contrast. [^89^Zr]Zr-DFO-ATG-031 produced far superior results, boasting a tumoral activity concentration of 16.2 ± 8.2 %ID/g at 120 h after injection and excellent tumor-to-background activity concentration ratios at the same time point: 21.3 ± 13.4, 6.4 ± 5.0, and 29.4 ± 22.0 for the blood, uterus or ovaries, and large intestine, respectively (Fig. 5).

## DISCUSSION

One of the most important recent developments in the study and management of endometrial cancer has been the replacement of the traditional type 1 or type 2 classification system with a 4-tiered approach defined by The Cancer Genome Atlas. In this system, cases are classified as POLE ultramutated (7% of cases), microsatellite instability (28%), copy-number high (39%), and copy-number low (26%) (22,23). Critically, these subtypes have clear prognostic and therapeutic implications. For example, copy-number high disease has the worst outcomes, whereas checkpoint inhibitors work best in disease with microsatellite instability (25). This emergent appreciation for the genetic diversity of endometrial cancer suggests that noninvasive molecular imaging (and PET in particular) could play a significant role in the management of the disease, specifically by helping clinicians identify patients who are likely to respond to targeted therapeutics. In this investigation, the PDXs spanned 3 molecular subtypes: microsatellite instability (P1), copy-number low (P2), and POLE ultramutated (P3). The subtype of P4 is unknown. The variability of antigen expression across subtypes is exemplified by MUC16, which is not appreciably expressed in P1 and P3 but has visible staining in P2 and P4. In contrast, HER2 and CD24 were expressed in all of the patient-derived samples. This, along with strong in vivo data, suggests that these 2 radiotheranostic targets could prove valuable across multiple subtypes of the disease.

The clear potential of HER2 and CD24 as molecular targets in endometrial cancer raises the question of how these 2 targets could be exploited for clinical nuclear medicine. The data presented herein suggest that [^89^Zr]Zr-DFO-trastuzumab and—perhaps even more so—[^89^Zr]Zr-DFO-ATG-031 could be useful for the diagnostic imaging of the disease. Along these lines, 2 possible applications are of particular interest: the delineation of potential lymph node metastases before surgical staging (thereby reducing operator and sampling biases in the selection of nodes for biopsy) and the detection of recurrent disease in the vaginal cuff after hysterectomy (a task for which current anatomic methods are inadequate).

It is more likely, however, that HER2- and CD24-targeted immuno-PET would be deployed as a companion imaging tool for molecularly targeted therapeutics. HER2 is a proven target for both immunotherapeutics (e.g., Herceptin; Genentech) and antibody–drug conjugates (e.g., Enhertu; Daiichi-Sankyo) (26). Indeed, the DESTINY-PanTumor02 trial identified trastuzumab–deruxtecan as a promising treatment for patients with HER2-positive endometrial cancer (27). The ZEPHIR trial has shown that immuno-PET with ^89^Zr-trastuzumab effectively delineated patients with breast cancer who would likely respond to a HER2-targeted antibody–drug conjugate (i.e., trastuzumab–emtansine), and our own work has demonstrated that HER2-targeted immuno-PET can identify both HER2-positive and HER2-low breast cancer, even in patients with HER2-negative primary disease (28). Taken together, these data suggest that immuno-PET with [^89^Zr]Zr-DFO-trastuzumab—or, alternatively, [^89^Zr]Zr-DFO-pertuzumab—could play an important role in identifying patients with endometrial cancer who are likely to respond to HER2-targeted therapies.

CD24 is, of course, a much more emergent therapeutic target than HER2. The antigen is overexpressed in a plethora of cancers and is believed to promote both cell migration and invasion. Furthermore, CD24 has been shown to have an important role in immune evasion, specifically through its interaction with the inhibitory receptor Siglec-10 on macrophages (16). Given these roles, it is not surprising that CD24 has emerged as a promising target for therapy. Indeed, several CD24-targeted treatments are currently in clinical trials, including 2 immunotherapeutics (i.e., IMM47 and ATG-031) and a CAR T-cell therapy (i.e., SWA11), and more are being explored at the preclinical stage (29,30). Although our study represents the first report of CD24-targeted nuclear imaging, previous work with programmed death ligand 1–targeting agents has ably demonstrated the potential of immuno-PET as a theranostic tool for use in immunotherapy (14). Thus, we believe that [^89^Zr]Zr-ATG-031 may be valuable in the clinic for the identification of patients likely to respond to CD24-targeted immunotherapeutics or antibody–drug conjugates.

Radiopharmaceutical therapy represents a third possibility for the application of HER2- and CD24-targeted radioimmunoconjugates in endometrial cancer. Admittedly, this is unlikely in the case of CD24, as the expression of the antigen by hematopoietic stem cells and lymphocytes raises the specter of significant hematotoxicity (16). The prospects are far more favorable, however, for HER2-targeting probes. Indeed, a wide variety of HER2-targeted radiotherapeutics—including those labeled with β-emitting (i.e., ^131^I and ^177^Lu) and α-emitting (e.g., ^225^Ac, ^212^Pb, ^211^At) nuclides—have been explored preclinically and clinically, yet none have been explored in the context of endometrial cancer (31,32). HER2-targeted radiopharmaceutical therapy may represent a path forward for the treatment of patients with disseminated or recurrent disease.

## CONCLUSION

This investigation illustrated the potential of HER2 and CD24 as radiotheranostic targets in endometrial cancer. We have shown that both antigens are abundantly expressed in endometrial cancer cells as well as patient-derived tissue samples representing several molecular subtypes of the disease. Furthermore, immuno-PET probes targeting [^89^Zr]Zr-DFO-trastuzumab and [^89^Zr]Zr-DFO-ATG-031 produced high tumoral activity concentrations and promising tumor-to-background activity concentration ratios in subcutaneous cell line–derived and patient-derived xenografts of the disease. In light of these data, we are currently working to determine the safety and efficacy of both antibody- and antibody fragment–based radiotherapeutics for the treatment of HER2-positive endometrial cancer. In addition, experiments are under way to explore the utility of [^89^Zr]Zr-DFO-trastuzumab and [^89^Zr]Zr-DFO-ATG-031 as companion theranostics in endometrial cancer alongside HER2-targeted antibody–drug conjugates and CD24-targeted immunotherapeutics.

## DISCLOSURE

Brian Zeglis reports funding from the National Institutes of Health (1R01CA281801, 1R01CA244327, and 1R01CA240963). Joni Sebastiano reports funding from the Tow Foundation. The content is solely the responsibility of the authors and does not necessarily represent the official views of the NIH. No other potential conflict of interest relevant to this article was reported.

## Notes

KEY POINTS **QUESTION:** Are HER2, MUC16, and CD24 promising radiotheranostic targets in endometrial cancer? **PERTINENT FINDINGS:** HER2 and CD24 are abundantly expressed in endometrial cancer cells as well as patient-derived tissue samples representing several molecular subtypes of the disease, while the expression of MUC16 is more sparing and variable. The immuno-PET probes [^89^Zr]Zr-DFO-trastuzumab (αHER2), [^89^Zr]Zr-DFO-AR96 (αMUC16), and [^89^Zr]Zr-DFO-ATG-031 (αCD24) exhibited a range of behavior in murine models of the disease. [^89^Zr]Zr-DFO-ATG-031 provided the highest tumor uptake and tumor-to-background contrast; [^89^Zr]Zr-DFO-trastuzumab produced moderate yet promising results; and [^89^Zr]Zr-DFO-AR9.6 yielded substandard images. **IMPLICATIONS FOR PATIENT CARE:** HER2- and CD24-targeted immuno-PET could prove valuable in the clinic as a theranostic tool to identify patients with endometrial cancer who are likely to respond to HER2- and CD24-targeted therapeutics. HER2 may also be a target for the radiopharmaceutical therapy of endometrial cancer.
